# Wild Animals in Captivity: An Analysis of Parasite Biodiversity and Transmission among Animals at Two Zoological Institutions with Different Typologies

**DOI:** 10.3390/ani14050813

**Published:** 2024-03-06

**Authors:** Lorena Esteban-Sánchez, Juan José García-Rodríguez, Juncal García-García, Eva Martínez-Nevado, Manuel Antonio de la Riva-Fraga, Francisco Ponce-Gordo

**Affiliations:** 1 Department of Parasitology, Faculty of Pharmacy, Complutense University, Plaza Ramón y Cajal s/n, 28040 Madrid, Spain; lorees01@ucm.es (L.E.-S.); jjgarc01@ucm.es (J.J.G.-R.); 2 Veterinary Department, ZooAquarium de Madrid, Casa de Campo s/n, 28011 Madrid, Spain; jgarciag@grpr.com (J.G.-G.); emartinez@grpr.com (E.M.-N.); 3 Veterinary Services, Parque Zoológico Faunia, 28032 Madrid, Spain; madelariva@faunia.es

**Keywords:** intestinal parasites, Protista, helminths, captive wild mammals, captive wild birds, zoological gardens, epidemiology, transmission risk

## Abstract

**Simple Summary:**

We have conducted a 10-year coprological study of animals housed in two zoological institutions with different housing conditions to assess parasite biodiversity and prevalence, their relationship with host class (mammal/bird), diet (carnivorous/omnivorous/herbivorous), and enclosure characteristics (soil type, isolation from wild fauna), and evaluated the risk of transmission to humans. A total of 4476 faecal samples from 132 mammal species and 951 samples from 86 avian species were examined, with 62.1% of mammal species and 12.8% of avian species testing positive. Statistically significant differences were found based on diet type; few carnivorous species were detected infected, primarily by nematodes, while many herbivorous and omnivorous species were primarily infected by protists. No statistically significant differences were observed based on soil type (artificial, natural, mixed) and isolation level (isolated/accessible). Several parasite species found in the study (*Entamoeba* spp., *Giardia* spp., *Balantioides coli*, *Trichuris* spp.) could potentially be transmitted between housed animals, wild fauna, and humans. Regular analyses of the animals and implementation and follow-up of health programs would minimise transmission risks between housed animals, wild fauna, and humans.

**Abstract:**

We have conducted a 10-year-long coprological study of the animals housed in two zoological institutions (ZooAquarium and Faunia, Madrid, Spain) to assess the parasite biodiversity, prevalence, and their relation with host class, diet, and enclosure type (soil type and level of isolation from wild fauna). A total of 4476 faecal samples from 132 mammal species and 951 samples from 86 avian species were examined. The results indicated that only 12.8% of avian species had parasites at least once during the study period, whereas 62.1% of mammal species tested positive. Predominantly, protists (*Entamoeba*, flagellates, and ciliates) and nematodes (mainly *Trichuris*) were identified in the findings. Carnivorous species were primarily infected by nematodes, while herbivorous and omnivorous species were mainly infected by protists. The number of infected herbivorous and omnivorous species was significantly greater than carnivorous species. Differences were observed based on soil type (artificial, natural, mixed) and isolation level (isolated/accessible), but these differences were not statistically significant. Several parasites (*Entamoeba* spp., *Giardia* spp., *Balantidoides coli*, *Trichuris* spp.) could potentially be transmitted between humans and some mammals and birds. Regular animal analyses and a personnel health program in the institutions would minimise transmission risks between zoo animals, wildlife, and humans.

## 1. Introduction

Parasites can affect their hosts both at an individual level (even causing their death) and at a population level, potentially affecting biodiversity by interfering in species competition, migration, and ecosystem stability [1]. The importance of parasites in the conservation of endangered host species is due to two circumstances: habitat degradation leads to increased contact between populations or species that are usually separated, easing the cross-transmission of pathogens, and the distribution of species populations in fragmented habitats leads to increased animal density, favouring disease outbreaks [2,3,4].

One of the most important objectives of zoological gardens is to contribute to the conservation of wild species, with special attention to those threatened or endangered in their natural habitats. Specific programs, such as the European Association of Zoos and Aquaria (EAZA) Ex situ programmes, are currently ongoing [5]. In the European Union, the importance of zoological gardens in education and species conservation is regulated by Directive 1999/22/EC, which, in the particular case of Spain, was transposed in 2003 into national law (31/2003). However, zoos could inadvertently serve as an opportunity for pathogens to be transmitted between individuals and species, given that the conditions mentioned above are present in zoo facilities: closer contact between different species and increased animal density. The occurrence of parasites in zoo animals could vary according to environmental conditions, management practices, disease prophylaxis, and treatment protocols [6,7]. Moreover, the physical characteristics of the facilities and the physiological status of the animals induced by captivity could contribute to the transmission of pathogens [8,9,10].

Despite the veterinary regulations in importing countries, zoo animals could be parasitised by co-imported parasites as well as other autochthonous species, and in some cases, cross-transmission with zoo personnel could occur [11,12,13,14,15]. However, although there are many studies on the prevalence of gastrointestinal parasites in zoo animals, only a few deal with the possible cross-transmission between housed and free-ranging animals or with the characteristics of the facilities [9,14,16,17,18]. In the present study, we investigated the biodiversity and host range of parasites infecting non-aquatic mammals and birds in two zoological institutions with different housing conditions during a 10-year period (2013–2022), compared the results between them, and evaluated the possibility of transmission between animals and humans.

## 2. Materials and Methods

### 2.1. Study Location and Host Species

This study was conducted from 2013 to 2022 in two zoos located in Madrid city (Spain): the ZooAquarium, situated in the Casa de Campo urban park, and Faunia Park, located within an urban area.

The ZooAquarium is organised into five main zones corresponding to different continents. In each region, animals are kept in groups or isolated by species based on their compatibility within enclosures of suitable size relative to the number of individuals. There is no crowding, and there are feeders, water sources, and hidden areas for resting. Flying birds are housed in open-air enclosures of adequate size. Mammals are in open-air natural areas delimited by water bodies and/or wood and metal fences; only a few species are animals in partially or totally enclosed installations limited by glass or metal fences and nets. The soil is natural and has grass in most sections; only in the case of large herbivores and some carnivores is there almost no grass. In some cases (i.e., the aoudads), the soil is concrete. The enclosures are encircled by paved pathways to accommodate visitor passage. In total, there are over 6000 animals of about 500 species from the 5 continents; the numbers vary over time depending on new acquisitions, deaths, and interchanges with other zoos.

Faunia Park is organised in ecosystems mostly recreated in closed installations; only in some cases are animals in open-air facilities. Depending on the zone and the species compatibility, animals are in open areas in direct contact with visitors’ pathways, in enclosures with wood or metal fences and nets, or in closed, isolated ambients recreating their natural habitat under controlled light and humidity conditions. There are more than 1200 animals of 152 species from 4 different ecosystems.

### 2.2. Sample Collection and Processing

Fresh faecal material was obtained from 83 species of terrestrial mammals and 64 species of birds at ZooAquarium and from 68 species of terrestrial mammals and 40 species of birds at Faunia Park. Nineteen mammals and eighteen bird species were housed at both zoos (for the purpose of this study, the Iberian eagle-owl, *Bubo bubo hispanus*, and the western Siberian eagle-owl, *Bubo bubo sibiricus*, are treated separately). They were classified as carnivores (including insectivores and scavengers), herbivores, or omnivores according to their main diet range. Mammal and bird scientific names follow the Mammal Diversity Database [19] and the International Ornithological Committee (IOC) World Bird List v.14.1 [20].

The samples were collected by the zookeepers early in the morning and kept in clean, new plastic recipients; they were transported to the laboratory 1–3 h after collection. Samples were usually processed upon arrival or kept at 4 °C until processed (maximum delay, 24 h). Individual samples were collected in some cases (i.e., when only one or a few individuals were in the group, from large animals, or in symptomatic or quarantined ones). All animals from the same species were sampled at the same time or in a two-week interval. In animals from large groups, faecal pools were collected. The results of the analyses were communicated to the zoo veterinarians, and they decided upon the correct treatment; in these cases, new samples were analysed after treatment to confirm their efficacy. Samples taken from animals that had received an antiparasitic treatment within the month before sampling were not included in this study.

Once in the laboratory, a macroscopic analysis of each sample was made, searching for the presence of parasitic structures. Faecal concentrates (following the formalin–ethyl acetate stool concentration technique) [21] were made, and the sediments were examined on temporary slides stained with Lugol’s iodine. Morphological features were measured and photographed with Olympus DP20 or Olympus DP23 cameras on an Olympus BX51 microscope (Olympus, Tokyo, Japan).

### 2.3. Parasite Biodiversity, Housing Conditions, and Feeding Habits

The possible relationship between the type of parasite life cycle (direct/indirect life cycle) and frequency of findings in each host species was investigated, taking into account the zoological institution, housing conditions, vertebrate class, and feeding habits as independent variables. The parasitological analyses were conducted for diagnostic purposes only, and a multifactorial analysis was not designed. Therefore, other environmental variables such as temperature, sunlight exposure, air or soil humidity, or rain were not considered during samplings. A time analysis was not performed as the samples from each host species were irregularly spaced over time, ranging from some weeks to more than one year between sampling a given species. Statistical comparisons were made using the IBM SPSS Statistics ver. 29 software (IBM Inc., New York, NY, USA).

Binary logistic regressions were conducted, considering the host species as “at least once infected” or ”never infected” as the dependent variable. Housing conditions were considered according to the type of soil and the level of isolation. The types of soil were categorised as natural (with/without natural vegetation; with natural drainage), artificial (cement base, with/without sand or wood shavings covering; without drainage or with drainage through artificial systems), or mixed (animals spending time in both natural and artificial soils, e.g., animals for exhibitions or with periods outdoors for environmental enrichment). In terms of isolation level, the animals were considered “isolated” when housed in enclosed spaces where access by wild fauna (small mammals like rodents or birds) was not possible or as “accessible” when uncontrolled access by wild fauna to the facilities was feasible. Regarding feeding habits, the host species analysed were classified as herbivorous, carnivorous (including ichthyophagous, insectivorous, and scavengers), or omnivorous, depending on their main diet type. For example, animals like lar gibbon (*Hylobates lar*) that may sporadically feed on animals but usually consume vegetables were considered herbivorous, while predators like wolf (*Canis lupus*) that may, in some instances, feed on vegetables were considered carnivorous.

## 3. Results

### 3.1. Overall Parasite Biodiversity and Prevalence

A total of 4476 faecal samples from mammals and 951 from birds (excluding those from repetitions after treatment courses) were collected and analysed from both zoos. Among them, 1333 samples from 82 mammal species and 63 samples from 11 avian species were found positive (Table 1). Parasites were found in 62.1% of the mammal species (82/132), while only in 12.8% of the avian species (11/86). The parasites found in mammals included protists (protozoa and chromists), trematodes, cestodes, and nematodes, while only one protozoan, one cestode, and several nematodes were found in birds (Table 2). In mammals, the higher number of host species found infected (mainly by protists) were herbivorous animals, while carnivorous hosts are the group with a lower number of species infected (Table 1 and Table 2). The morphological characteristics of eggs/cysts/oocysts often do not allow for differentiation between species. In cases where morphologically similar genera or species infect the same or related host species, the parasites were identified using group names (e.g., trichomonads, trichostrongylids) or as spp. (e.g., *Trichuris* spp.). Findings resembling the amoebae species *Entamoeba bovis* Liebetanz 1905, *Entamoeba polecki* Prowazek 1912, *Entamoeba coli* (Grassi 1879), and *Entamoeba muris* (Grassi 1879); the ciliate *Balantioides coli* (Malmstem 1857); and the cestode genus *Raillietina* Fuhrmann 1920, were identified as taxon-like.

**Figure 1:**
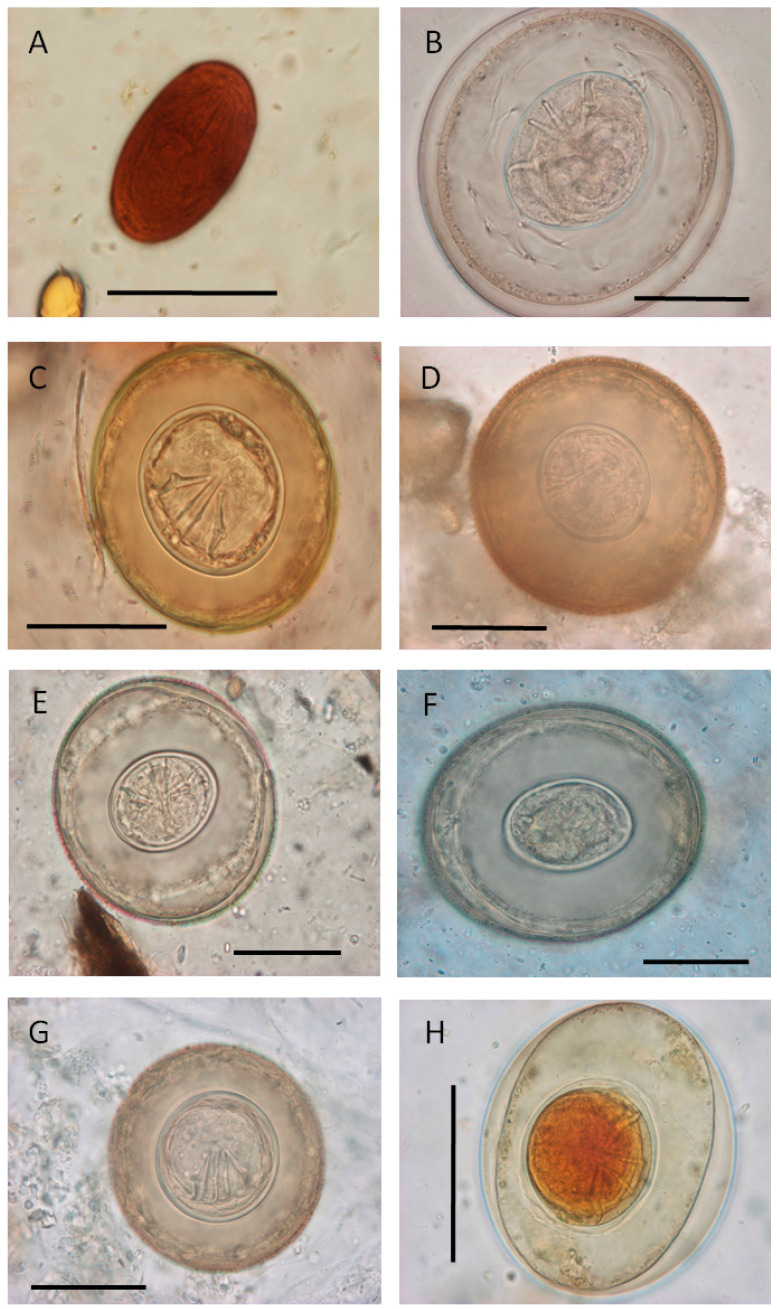
Eggs from indirect life-cycle parasite species found in the samplings. (**A**) Unidentified trematode egg resembling *Dicrocoelium* (Dujarding 1845) egg from the sun bear (*Helarctos malayanus*). (**B**–**H**) Cestode eggs. (**B**) *Raillietina*-like eggs from the helmeted guineafowl (*Numida meleagris*). (**C**) Unidentified egg from the red deer (*Cervus elaphus*); (**D**) unidentified egg from the yak (*Bos grunniens*); (**E**) unidentified egg from the South American tapir (*Tapirus terrestris*); (**F**) unidentified egg from the hippopotamus (*Hippopotamus amphibious*); (**G**) unidentified egg from common brown lemur (*Eulemur fulvus*); (**H**) unidentified egg from the mandrill (*Mandrillus sphinx*). Scale bars: 30 µm.

The biodiversity and prevalence of the parasites found in each host species are given in Table 3, Table 4, Table 5 and Table 6. All parasites found were of the direct life cycle, except for the unidentified trematode eggs found in bears and the cestodes found in several mammal species in the ZooAquarium and in one bird in Faunia (Figure 1).

The protists were the most frequently identified parasite group and the only one found in 34 mammal and 3 avian species in ZooAquarium and in 15 mammal species in Faunia (Table 2, Table 3, Table 4, Table 5 and Table 6). Helminth-only infections were found in six mammal and four avian species in ZooAquarium and in five mammal and four avian species in Faunia. Finally, both protists and helminths were recorded in 20 mammal species in ZooAquarium and 5 mammal and 1 avian species in Faunia. The host species with the higher parasite biodiversity were the dama gazelle (*Nanger dama*) in mammals and the helmeted guineafowl (*Numida meleagris*) in birds, which were infected (not simultaneously) by up to 8 and 4 different parasite species, respectively. Single parasitisms were found in most positive samples; in polyparasitisms, the maximum number of parasite species causing a simultaneous infection was 4 (in the dama gazelle). The most common parasitic genera found were *Entamoeba* (in 44 host species in ZooAquarium and 13 in Faunia), *Balantioides* (in 14 host species in ZooAquarium and 3 species in Faunia), and *Trichuris* (in 14 and 9 host species in ZooAquarium and Faunia, respectively).

#### 3.1.1. Avian Hosts

The differences in the number of species analysed correspond to the collection design by the management of the zoological institutions. By feeding type, the number of samples analysed at ZooAquarium is proportional to the number of bird species; in the case of Faunia, the number of samples from carnivorous species is proportionally much lower (Table 1) because omnivorous and herbivorous species are kept in groups, making it easier to find valid samples for analysis than in the case of carnivorous species, which must be housed individually in most cases.

Only nematodes, including capillariid and ascarid eggs, were identified in carnivorous species (in birds of prey at ZooAquarium and Faunia, as well as in gruiformes specifically, the common crane, *Grus grus*, at Faunia) (Table 4 and Table 6). In omnivorous species, only protists (*Entamoeba* spp. and *B. coli*) were detected at ZooAquarium (Table 4), while nematodes (capillariids and ascarids) and cestodes (with one observation of *Raillietina*-like eggs in the helmeted guineafowl) were exclusively found at Faunia; additionally, *E. gallinarum* was detected in the helmeted guineafowl at Faunia (Table 6).

#### 3.1.2. Mammalian Hosts

Almost all herbivorous species were infected but generally exhibited low parasite biodiversity. The most prevalent parasites in herbivorous mammals were amoebae (*Entamoeba*) (Table 2)*,* which were found in nearly all hoofed animals (except equids), suids, and macropodids (the yellow-footed rock-wallaby, *Petrogale xanthopus*, and Bennett’s wallaby, *Notamacropus rufogriseus*) (Table 3 and Table 5). The *Entamoeba* cysts found in these hosts were uninucleated in all cases, except in two samples from the dama Gazelle and one from Bennett’s wallaby, where eight unidentified cysts were present. The one-nucleated *Entamoeba* cysts were of two types; those from hoofed animals were small (4–10 µm in diameter) and were identified as *Entamoeba bovis*-like, while those from suids and the tapir were larger (15–20 µm in diameter) and were identified as *Entamoeba polecki*-like. The species from macropodids were not identified.

*Giardia* infection in herbivores was rare; cysts were detected on a few occasions in the dama gazelle, the red river hog (*Potamochoerus porcus*), and the Patagonian mara (*Dolichotis patagonum*) in ZooAquarium (Table 3), and in the Southern red muntjac (*Muntiacus muntjak*), the Patagonian mara, the Brazilian porcupine (*Coendou prehensilis*), and the aardvarks (*Orycteropus afer*) in Faunia (Table 5). Among the ciliates from herbivorous hosts, *B. coli*-like cysts were found in suids, the sitatunga (*Tragelaphus spekii*) and the South American tapir (*Tapirus terrestris*); the identifications were based on the cyst size (about 40 µm in diameter). In camels (*Camelus bactrianus* and *Camelus dromedarius*), the cysts were of greater diameter (around 80 µm) and were identified as belonging to *Buxtonella cameli*. Entodiniomorphid ciliates were frequently found in equids, elephants, and rhinoceronts.

Helminth infections in herbivorous species were mainly caused by trichostrongylids and trichurids in the dama gazelle and in the dorcas gazelle (*Gazella dorcas*), and by trichurids/capillariids in some hoofed animals and in rodents (the Patagonian mara). The identification of eggs belonging to genera *Trichuris* or *Capillaria* was based on the appearance of the eggshell (thick and smooth in *Trichuris*, striated in *Capillaria*). Cestode eggs (Figure 1) were found only on isolated occasions in the reed deer (*Cervus elaphus*), the hippoptamous (*Hippopotamus amphibious*), the South American tapir, and the Patagonian mara, all of them in ZooAquarium.

Among the omnivorous species, primates were the group with the greatest number of species infected, mostly by protists (Table 2); the mandrill was the species harbouring the widest range of parasites (*Entamoeba* spp., *Chilomastix* spp., *B. coli*, *Trichuris* spp., and an unidentified cestode), and *Giardia* cysts were found only in lemurs (ring-tailed lemur, *Lemur catta*, and Mayotte lemur, *Eulemur fulvus*) (Table 3 and Table 5). Two different types of *Entamoeba* cysts were found in primates: one nucleated cyst identified as *E. polecki*-like and eight nucleated cysts identified as *E. coli*-like. The *E. polecki*-like cysts found in this study were clearly larger (10–16 µm in diameter) compared to those found in hoofed animals and similar to those identified as *E. polecki*-like in suids and tapirs.

In Ursidae, nematodes (*Baylisascaris*) were detected in the brown bear (*Ursus arctos*); trematode eggs (Figure 1) were found in one sample from the sun bear (*Helarctos malayanus*).

Very few carnivore species were found infected, typically by nematodes (Table 2); only the giant anteater (*Myrmecophaga trydactila*) was found infected by nematodes and protozoa (capillariid eggs, four-nucleated *Entamoeba* cysts, *B. coli*-like cysts, and trichomonad flagellates), while the bushdog (*Spheotos venaticus*) only by coccidia. Cestode and trematode eggs were found once in several species at ZooAquarium (Table 3).

### 3.2. Biodiversity and Prevalence in Relation to Feeding Habits and Housing Conditions

Before examining the obtained results, it is necessary to consider that the unequal number of samples analysed within some of the considered categories (Table 7) can introduce biases in the estimation of regression coefficients, wider confidence intervals, and the statistical significance of the coefficients. In the latter case, the significance would probably not be affected when “clear” significant or non-significant statistical values were obtained (i.e., *p* > 0.100 or *p* < 0.001), but in those cases where we have found *p*-values in the range 0.010–0.050, the interpretation of the associations should be taken with care and generalising results to broader populations of zoo animals may be challenging.

The distribution of host species according to the type of zoological institution, vertebrate class, housing conditions (isolation level and soil type), and feeding habits is shown in Table 7. None of the avian species analysed were housed in isolated spaces in either of the zoos.

The initial analysis involved five independent variables (zoological institution, host class, soil, isolation, and feeding habits) (Table 8). The Hosmer and Lemeshow X^2^ test (HLt; *p* = 0.003) was significant, indicating that the regression model did not fit the observed data well. The model explained 47.4% of the variation (Nagelkerke R^2^ = 0.474) and 82.0% of the samples would be correctly classified. When soil type and host class (which were highly and statistically correlated with the other variables) were removed from the analysis, the HLt was non significant (*p* = 0.807). However, both the percentage of data variation explained by the model and the percentage of samples correctly classified decreased (R^2^ = 0.275; 71.8% correct sample classification). Under these circumstances, we chose to use the model with all the independent variables to analyse the importance and influence of each one.

The variables that exhibited higher importance for interpreting the data were host class (Wald test, *p* < 0.001) and feeding type (*p* < 0.001) (Table 8). There was no statistically significant increase (*p* = 0.109) in the probability of finding infected hosts in either zoological institution, although this probability was slightly lower in Faunia than in ZooAquarium (B coefficient = −0.649). The number of host species found infected was nearly identical when the animals were kept in “natural” and “artificial” types of soil and lower in mixed soil; however, these differences were not statistically supported (*p* = 0.301). The probability of animals in enclosed spaces being infected was 1.326 times lower than those kept in open areas, although these differences were not statistically supported (*p* = 0.456). Regarding feeding type, the probability of positive samples in omnivorous and herbivorous species was similar between them and markedly higher (1.4581 and 1.911 times, respectively) and statistically significant (*p* = 0.003 and *p* < 0.001, respectively) than in carnivorous species.

In relation to host class, the probability of positive samples was 2.1 times lower in avian species than in mammalian ones. As the conditions in which mammals and birds are housed and fed differ, we conducted separate analyses for each group (Table 9 and Table 10). In both cases, the regression models fit the observed data well (HLt = 0.944 and 0.860 for the mammalian and avian data, respectively); the important increase in the standard error of the constant in the equation is a consequence of the small number of data available in some categories (Table 7). The percentages of data variation explained by the models were similar (46.1% for mammalian data) or lower (27.8% for avian data) compared to the combined analysis, and the percentages of samples correctly classified (77.5% in mammals, 90.4% in birds) were similar. In the analysis of the avian samples, none of the variables had a statistically significant effect at the *p* < 0.05 level. However, feeding habits approached this limit (*p* = 0.060) due to the greater number of omnivorous species found infected compared to carnivorous ones, though not at the *p* < 0.01 level (*p* = 0.018). In mammals, a similar situation was observed regarding the number of positive species in each zoological garden (1.245 lower in Faunia than in ZooAquarium; *p* = 0.019); the only variable that was clearly significant was feeding habits, with carnivorous species being the less infected group.

## 4. Discussion

In this study, we present the results obtained from the parasitological analysis of birds and mammals from two zoological facilities with different topologies. While other published studies on zoo animal parasites focused on hosts belonging to a small group of taxonomically related species [22,23,24] or based on their feeding habits [25,26,27], the present work stands out for the number and zoological diversity of the host species analyzed, including 15 orders of mammals and 23 of birds. The objective of this study is to assess the importance of various factors in the transmission and dissemination of parasites among zoo animals, as well as in relation to humans (zoo personnel and visitors).

With a few exceptions noted below, all the intestinal parasites identified in this study have been previously described in captive animals housed in zoological gardens [9,26,28,29,30,31,32]. We should note that this study had some limitations: no specific stainings were performed to detect the presence of *Cryptosporidium* oocysts or microsporidia spores, and the presence of *Blastocystis* was not routinely investigated. These parasites are mostly considered in specific studies, but only *Cryptosporidium* is sometimes investigated in broad host-range studies in zoos [6,7,32,33,34]. Another limitation is that no genetic studies have been conducted except in specific cases, thus preventing the precise identification of the species found.

### 4.1. On the Parasite Epidemiology, Biodiversity, and Species Identification

#### 4.1.1. Avian Hosts

In our study, the number of infected host species was low and similar in both zoos (10.9–12.5%), although the number of positive samples was clearly higher in ZooAquarium (53.1%) than in Faunia (7.0%). In a general comparison, these values are within the range of prevalences reported in other studies, with nematodes being the most frequently mentioned group [35].

The hosts we found parasitised are mainly Accipitriformes and Galliformes; also, Gruiformes, Picirformes, Strigiformes, and ratites (Struthioniformes and Rheiformes). Galliformes are the group of zoo birds most commonly reported to be parasitized [9,29,31,36,37,38,39]. There are few studies on birds of prey (Accipitriformes, Falconiformes, and Strigiformes) in which capillariids and coccidia are the parasites most frequently found [39,40]. In our study, we did not find parasites in Psittaciformes or Passeriformes, groups that other authors found nematodes (mainly ascarids and trichurids) and coccidia with prevalences of up to 40% in some cases [9,28,29,30,32,36,39,41,42].

In Struthioniformes and Rheiformes, although parasitic biodiversity can be high in captive birds, especially the ostrich (*Struthio camelus*) [43], our findings were scarce and limited to protists in the ostrich and the rhea (*Rhea Americana*), similar to other results in Spain [37] and Brazil [7]; however, also in Brazil, nematodes (ascarids) were reported in the rhea and the cassowary (*Casuariius casuarius*) [18]. Coccidia and *Capillaria* were reported in the cassowary [39]. In Serbia, a low number of positive samples were reported in the emu (*Dromaius novaehollandiae*), the ostrich, and the rhea (29% in total), although the parasitic biodiversity was much higher, including unidentified ciliates (probably *B. coli*), unidentified ascarids, and strongyles (probably *Lybiostrongylus* Lane 1923, misidentified as *Strongyloides* Grassi 1879) in the ostrich, and *Capillaria* in the rhea [44]; similar results were obtained in the ostriches in zoos in Nigeria [38].

The vast majority of parasites reported in previous studies are species with a direct life cycle; however, unidentified trematodes were also occasionally reported [9,36,39]. The cestode eggs found in our study in the helmeted guineafowl may correspond to *Raillietina*, although since the morphology of the eggs is similar to that of *Hymenolepis* Weinland 1858 eggs, the identification is tentative. Overall, depending on the cestode species and the intensity of the infection, this can be asymptomatic or lead to diarrhoea and intestinal lesions [45,46].

In our study, the most common group of nematodes was the capillariids, found in 7 species of birds (Table 4 and Table 6). This generic term includes the genera *Baruscapillaria* Moravec 1982, *Capillaria*, *Echinocoleus* López-Neyra 1947, *Eucoleus* Dujardin 1845, *Ornithocapillaria* Barus and Sergeeva 1990, *Pterothominx* Freitas 1959, and *Tridentocapillaria* Barus and Sergeeva 1990 [47]. In cases where species from more than one genus were described in the corresponding host, we used the term “capillariid” (e.g., in the Steller’s sea eagle, *Haliaeetus pelagicus*, where *Eucoleus dispar* Dujardin 1845 and *Capillaria tenuissima* (Rudolphi 1809) were described). We identified the eggs found in the Harris’s hawk (*Parabuteo unicinctus*) as *Trichuris* based on their morphology; however, we have not found any species of *Trichuris* or *Capillaria* described in this bird, so the possibility of spurious parasitism cannot be ruled out. Pérez-Cordón et al. [33] identified *Trichuris* in some of their samples but without specifying the host species. The clinical significance of these parasites depends on their location in the host and the intensity of infection; in fatal cases, there may be no clinical signs, or they may be nonspecific (e.g., diarrhoea, anorexia, weakness) [47].

The other group of nematodes found in birds includes the ascarids, found in Galliformes (*Ascaridia*/*Heterakis*) and Accipitriformes (*Porrocaecum*). The genus *Porrocaecum* includes two cosmopolitan species that affect birds of prey, *Porrocaecum angusticolle* (Molin 1860) and *Porrocaecum depressum* (Frölich 1802); in both cases, infections typically do not produce clinical signs or severe disease in birds, although they may occasionally lead to death [48]. On the other hand, several species of *Ascaridia* and *Heterakis* have been reported in the helmeted guineafowl and the red junglefowl/chicken (*Gallus gallus*) [49], with the eggs of species from both genera being very similar; for this reason, we considered it preferable to identify them as *Ascaridia*/*Heterakis*. The symptoms produced by these nematodes are nonspecific and can result in the death of the affected animal. In wild populations, infections by these parasites can have a negative impact on development, lower host body condition, and worse rates of survival and reproduction [49].

In the present study, we did not find coccidia in the bird samples, although this is generally the most frequently encountered group of protists, with a range of 9–100% of positive samples when present [9,18,31,39]. The most common genera of coccidia in birds are *Eimeria* and *Isospora* Schneider 1881 [50,51], although *Caryospora* Leger 1904, *Tyzzeria* Allen 1936, and *Wenyonella* Hoare 1933 have also been reported in birds [52]. In general, the symptoms caused consist of diarrhoea, enlarged abdomen, loss of appetite and weight, and even death.

The only protists identified in birds in this study were ciliates (*B. coli*) and amoebae (*Entamoeba* spp.). *Balantioides coli* were found only in the ostriches, as reported in other studies [7,38,44]. Additionally, three species of *Entamoeba* were identified, forming uninucleated cysts (in the ostriches), tetranucleated cysts (in the rheas), and octonucleated cysts (in the chickens and the guineafowl). Uninucleated cysts are commonly found in ratites, especially the ostriches [43], where the species *Entamoeba struthionis* Ponce-Gordo et al. 2004 was described [53,54]. However, in other studies, *Entamoeba* was not reported, but other protists (ciliates) were recorded [38,44]. In the rhea, *E. polecki* and *Entamoeba suis* Hartmann 1913 were identified through genetic analysis [55,56], as well as an unidentified species forming octonucleated cysts [43]. The tetranucleated cysts found here were genetically analysed and correspond to *Entamoeba dispar* Brumpt 1925 [57]. In captive emus in Brazil, uninucleated cysts identified as *Entamoeba* spp. were also found [58]. Regarding *Entamoeba gallinarum* Tyzzer 1920, a species forming octonucleated cysts described in galliforms, there are few studies reporting its presence, and always with a low prevalence [9,59].

#### 4.1.2. Mammalian Hosts

The number of species found infected varied according to the zoological institution, being overall and by animal group (according to their diet) higher in ZooAquarium than in Faunia. However, the values found in ZooAquarium are within the range of results published by other authors [25,31,32,60], and compared to other European zoos, the values are also similar or even lower [26,34,44,61]. These overall data, however, require a more detailed analysis as there are significant differences depending on the mammalian group considered.

Among carnivorous animals, we only found positive samples in some species of the order Carnivora and in the giant anteater (order Pilosa) (Table 3 and Table 5). In species of the order Carnivora, the vast majority of findings occurred only once or twice over the 10 years of sampling, and except for four positive samples for *Capillaria*, the parasites now found do not correspond to those generally detected in other studies, which report ascarids, whipworms, and strongyles [13,25,31,34,44,60,62].

The highest number of infected species, and positive samples, was observed in herbivores and omnivores. Almost all species of Artiodactyla tested positive for amoebas (*Entamoeba*), and those of Perissodactyla, for ciliates. In some hoofed animals, cestode eggs were found (the species could not be determined), and some species also showed persistent nematode infestation by *Trichuris*, *Capillaria*, and/or trichostrongylids; however, the overall helminth prevalence relative to protists was lower. In other studies, helminth infections in hoofed animals were predominant [6,37].

Non-human primates (NHP) are one of the mammal groups commonly studied in zoo animal research [35]. The parasites typically reported in these animals include *Entamoeba*, *Giardia*, and *Trichuris* [3,7,31]; other protists (*Giardia*, coccidians, *Cryptosporidium*, ciliates) and helminths (such as strongylates, ascarids, oxyurids, and spirurids) were also occasionally documented [7,23,30,34,44,61,63,64,65]. Similar to hoofed animals, helminths were typically reported in NHPs with a higher prevalence than protists [7,31,32,34]; however, in some studies, protists (mainly *Entamoeba*, *Giardia*, and ciliates) were more common [22].

By parasite group, *Entamoeba* spp. were the most frequently encountered protozoa in herbivorous and omnivorous mammals. In carnivores, *Entamoeba* cysts were found in the giant anteater, and they were genetically identified as *E. dispar* [57]. The remaining species found belong to either the *E. polecki* group (forming uninucleated cysts) or the *E. coli* group (forming octonucleated cysts). The species *Entamoeba ovis* Swellengrebel 1914 and *E. bovis* (forming uninucleated cysts) were described in various ruminant species, but due to the difficulty in morphological differentiation (the size ranges overlap), we have preferred to identify them as *E. bovis*-like [66]. The uninucleated cysts observed in suids are larger, but there are several morphologically indistinguishable species that can infect them (*E. polecki*, *E. struthionis*, and *E. suis*). In NHPs, the one-nucleated cysts are commonly identified as *Entamoeba chattoni* Swellengrebel 1914 [67,68,69,70,71] or as *E. polecki* [72,73,74], both morphologically indistinguishable. Therefore, in suids and NHPs, we identified the uninucleated cysts as *E. polecki*-like [66]. The eight-nucleated cysts found in NHPs would correspond to *Entamoeba coli*, but since it is actually considered a species complex [75], it would be best to identify the findings as *E. coli*-like. In general, the *E. bovis*-like, *E. polecki*-like, and *E. coli*-like species are considered non-pathogenic, although [76] reported a case of symptomatic infection in humans by *E. polecki*, [77] suggested an association between the presence of *E. bovis* and diarrhoea in cattle, and Coke et al. [78] reported a fatal case in which unidentified *Entamoeba* and *Acanthamoeba* Volkonsky 1931 were found in gastric ulcers in an 11-month-old female giant anteater. Except for the *E. bovis*-like species, all other *Entamoeba* spp. from zoo mammals can infect humans.

Ciliates are the second most commonly encountered group of protists in our study in terms of findings and infected hosts; however, they are usually not reported except in specific studies. There is a great diversity of ciliates described in Artiodactyla, Perissodactyla, and Proboscidea, mostly corresponding to species of the orders Entodiniomorphida and Vestibuliferida [79,80,81,82,83,84]. Since the identification of different genera and species requires specific staining methods, and most of these species are considered commensal/endosymbionts, we made a generic identification of the findings in the hippopotamus, equids, rhinoceroses, and elephants as “endosymbiotic ciliates”. From a human and animal health viewpoint, the ciliate species with greater relevance are vestibuliferid ciliates (*Balantioides* and *Buxtonella*) from some hoofed animals (camels, suids, and tapirs) and from NHPs. They can be transmitted to humans (at least *B. coli*) and have been considered by several authors as potentially pathogenic for their hosts [85,86,87,88]. Based on cyst size, our findings in some hoofed animals (the sitatunga, *Tragelaphus spekii*, the moose, *Alces alces*, the collared peccary, *Dicotyles tajacu*, the red river hog, and the pigs) and in NHPs would correspond to *B. coli*, while in large bovids (the European bison, *Bison bonasus*, the yak, *Bos grunniens*, and the African buffalo, *Syncerus caffer*), the ciliate was identified as *Buxtonella sulcata*. The species infecting camels, usually reported as *B. coli*, is *Buxtonella cameli* [88]. The identification of *B. coli* of the NHP cysts should be considered tentative, as an unnamed *Buxtonella* sp. whose cysts are similar to those of *B. coli* could also infect NHPs [87]. When these protists are reported in studies on zoo animals, their prevalence is highly variable, ranging from 10–22% in hoofed animals to 9.5–80% in NHPs (the macaques, the chimpanzees, and the orangutans) [6,7,32]. Also, in NHPs, in the present study, we found some gorilla samples positive for *Troglodytella*, a rare finding in zoo populations. Our findings occurred after a new gorilla from an England zoo was introduced to the group. While this ciliate is common in wild African great apes (and *B. coli* is rare), the different diet in captivity leads to the opposite situation in zoo animals and even to the disappearance of *Troglodytella* [89,90].

*Giardia* cysts were observed in several host species. *Giardia duodenalis* is considered a species complex, with its genetic variants typically regarded as assemblages [91]. A recent proposal for taxonomic revision [92] has been made to assign these assemblages to defined species. The new findings in NHPs (in the common brown lemur, *Eulemur fulvus*, and the ring-tailed lemur, *Lemur catta*) would correspond to the *G. duodenalis* Stiles 1902 assemblage B/*Giardia enterica* Grassi 1881, according to previous records [93]. Maesano et al. [61] also found *Giardia* in the ruffled lemur (*Varecia variegata*), the gorilla (*Gorilla gorilla*), and the capuchin monkey (*Sapajus apella*), although they did not specify the species. According to the recent taxonomic proposal [92], the findings we made in other mammals (hoofed animals, the crested porcupine, *Hystrix cristata*, and the red river hog) may correspond to *G. duodenalis*, *Giardia intestinalis* (Lambl 1859), or *G. enterica*; the findings in the aardvark cannot be presumptively assigned to any of the newly (re)described species.

The finding of tapeworm eggs in species housed in zoos is rarely reported [6,64]. In our study, the findings were occasional, and the morphology of the eggs did not correspond to that of the tapeworm species cited in the corresponding hosts, suggesting that they could be spurious parasitoses. The presence of eggs resembling *Hymenolepis* in Madagascar lemurs was mentioned [94], but no species were described.

In nematodes, the most frequent findings corresponded to *Trichuris* and *Capillaria* eggs. We found capillariid eggs in different anteater individuals at the ZooAquarium, but it is not possible to identify the genus because there are no previous descriptions in this host species; Diniz et al. [95] indicated that 28% of the samples they analysed were positive for *Trichuris*, although they did not provide specific details or indicate the possible species.

Several species of *Trichuris* could infect hoofed animals (*Trichuris ovis* (Abidgaard 1795), *Trichuris discolor* (von Linstow 1906), and *Trichuris skrjabini* Baskakov 1924), so it is not possible to make a specific identification with the available data. The *Capillaria* eggs in the fallow deer (*Dama dama*) could correspond to *Capillaria bovis* (Schnyder 1906) [96]. In NHPs, spurious parasitosis would explain occasional findings in the mandrill (*Mandrillus sphinx*), the Müller’s gibbon (*Hylobates muelleri*), and the lemurs; however, the repeated findings in the colobus (*Colobus guereza*) and the baboons (*Papio* spp.) would indicate true infections. The species in NHPs are typically identified as *Trichuris trichiura* (Linnaeus 1771) [97], but *Trichuris colobae* Cutillas et al. 2014 was also described in the colobus [98], *Trichuris ursinus* Callejón et al. 2017 in the baboon [99], and *Trichuris lemuris* Rudolphi 1819 in the lemurs [100]. In other studies in zoo NHPs, *Trichuris* spp. were found with prevalences between 20 and 100% [34,101]. At least *T. trichiura* can be transmitted to humans. Mild infections are usually asymptomatic, but fatal cases have been described in NHPs [102].

Regarding the trichostrongylids, the only findings occurred in hoofed animals; NHP samples were always negative. The morphological and size similarity of the eggs found in hoofed animals makes it difficult, if not impossible, to differentiate the eggs of different genera, so identifications are usually conducted generically as “strongyle type” or “trichostrongylids” [32,103,104,105]; if anything, *Nematodirus*, due to its size, can be identified separately [61,104]. Depending on the helminth species and the intensity of the infection, animals may be asymptomatic or suffer from gastrointestinal symptoms (especially in trichostrongylid infections); *Nematodirus* can be highly pathogenic and cause death within a few days after the onset of symptoms [106].

The ascarid eggs found in carnivores belong to *Baylisascaris*. In the Brown bear, the species could correspond to *Baylisascaris transfuga* (Rudolphi 1819), which was identified in wild host species in Europe and Asia [105,107]. In the case of the striped skunk (*Mephitis mephitis*) samples, it could correspond to *Baylisascaris columnaris* Leidy 1856, which was detected in other European zoos [108]. We did not find *Toxocara*/*Toxascaris* infections despite their prevalence potentially being high in zoo animals [109]. In equids, *Parascaris equorum* can be recorded in zoo animals with a low prevalence, usually below 15% [32,33,105,110].

### 4.2. Effect of Housing Conditions

Considered collectively, the results obtained in both zoological parks show parasitic prevalences (Table 1) lower than those observed in many other studies [6,24,33,37,44,111,112]. The differences between the results from different zoos can be attributed to a multitude of factors such as animal density, their immune status, the design of the facilities, perimeter barriers, or preventive medicine programmes (staff control, biosecurity measures, cleanliness, routine monitoring of the animals) [35]. In the present case, the animal density and the preventive medicine programmes were the same, as both institutions belong to the same leisure park operator and have the same protocols; the only differences between the two centres are the location and design of the facilities and the animal collection housed. In other comparative studies between zoos [34,104], differences among centres were attributed to the type of facility, the possibility of herbivore grazing, and the frequency of faeces collection and cleaning. Other important factors include possible water or food contamination (animal carcasses for carnivores, grass and herbaceous material for herbivores) [37,113,114]. In the present study, control over water and food provided to the animals is similar in both zoos, so the differences in the observed results between them must have another origin.

We have observed that some parasites appeared more frequently (those with high detection percentages in Table 3, Table 4, Table 5 and Table 6), but there was not an apparent direct relationship with population size. For example, in ZooAquarium, there were numerous groups of dama gazelles and a small group of fallow deer; both species commonly had *Entamoeba bovis*-like infections (70–80% of positive samples), but nematode infections were occasional (e.g., *Trichuris* spp. was found in 15–16% of gazelle samples and *Capillaria* in 5% of fallow deer samples).

Although the results obtained in our study concerning accessibility by uncontrolled fauna are not statistically significant, the overall presence of parasites was 1.3 times higher in species in accessible environments compared to those in controlled environments. In this regard, there are no major differences in the general typology of bird facilities between ZooAquarium and Faunia. All birds are in accessible environments, and the incidence of parasitised species is similar in both centres; the slightly higher (statistically non-significant) incidence of parasitism in Faunia may be due to the greater presence of multi-species installations and aviaries, which would facilitate transmission among birds. However, in the case of mammals, the likelihood of a species harbouring parasites in accessible environments was more than 19 times higher than in isolated ones, but no statistically significant differences were found between ZooAquarium and Faunia (where there are a greater number of species in controlled environments) most likely due to mathematical issues (highly unbalanced number of cases in each factor combination; see Table 7). It has been proposed that the possible existence of microclimates within the parks may provide the necessary humidity and temperature for the survival of some pathogens [115]. However, this circumstance would not explain the differences between ZooAquarium and Faunia; while ZooAquarium has a greater number of interior concrete sleeping quarters, where eggs/cysts/oocysts can be maintained in more humid environments and protected from solar radiation, Faunia has a greater number of controlled, enclosed installations without direct sunlight.

One of the likely most important factors to consider is the entry of parasites transported by carriers (i.e., insects) or transmitted by local wildlife that enter the zoo in search of food [14,16,18]. This would allow for similar prevalences of direct-cycle parasites in animals from outside and inside the park [9,10,17]. In the case of parasites with an indirect life cycle, the uncontrolled entries of infected intermediate hosts can lead to the occurrence of infections by adult cestodes in some cases, while the entrance of infected adult hosts (i.e., mesocarnivores) into reserved areas could result in the emergence of larval cestodiasis, which can be lethal for zoo animals [16]. ZooAquarium and Faunia are located close to each other in the same city (about 15 km apart in a straight line), so, a priori, there is not a significant difference in the potential wild animals that may introduce parasites into both centres. The climatic conditions are also similar, and these do not seem to differentiate the results between different zoos [115]. However, ZooAquarium is situated within the largest urban park in Madrid, where sheep and goats graze during certain times of the year, and there is a greater presence of wildlife in the surrounding environment compared to Faunia, which is situated in a more urban setting. In the case of cestode eggs found in some mammals at the zoo, regardless of whether they are genuine or spurious parasitosis, their origin should be linked to infected animals from the outside (intermediate hosts with larvae, adult hosts excreting eggs in faeces) that entered into the zoo facilities. In addition to local wildlife, the public can also introduce parasites (e.g., eggs, cysts, or oocysts on footwear) from the outside to the inside of the facilities. An effective way to prevent contagion would be to limit public and wildlife access to animal facilities or keep the hosted animals in isolated environments, both circumstances being more prevalent in Faunia than in ZooAquarium installations.

The sanitary control of food is another important factor. In carnivorous species, food is often frozen for a few days before use, which helps kill tissue forms of protists and helminths. However, this pretreatment is not usually carried out with vegetables to maintain their appearance and palatability, and if they are not processed with extensive washing, there is a high probability of transmission of cysts/oocysts/eggs. Several studies have shown the contamination of fruits and vegetables sold to consumers with parasitic structures (cysts, oocysts, and eggs) in countries across all continents [116,117,118,119]. In Europe, Federer et al. [120] studied the presence of taeniid eggs in the vegetables and fruits fed to gorillas in Basel Zoo (Switzerland). Despite the vegetables being of high quality, processed at high hygienic standards, and pre-washed by the farmer, the authors later identified the DNA of several taeniid species (*Taenia crassiceps* (Zeder 1800), *Taenia hydatigena* Pallas 1766, *Taenia multiceps* Goeze 1782/*Taenia serialis* (Gervais 1847), *Taenia saginata* Goeze 1782, and *Hydatigera taeniaeformis* (Batsch 1786)) in wastewater obtained after the routine processing of the food in the zoo food preparation station. The risk exists, but the problem is that there are not always well-established, standardised, or validated methods for detecting parasites in food [121].

Another possible cause of the greater impact generally experienced by herbivorous and omnivorous species compared to carnivores may be their feeding behaviour and the substrate on which they feed. The herbivorous/omnivorous species directly ingest food from the ground or, in the case of primates, using their hands, which are also used for locomotion; thus, contact with parasite transmission forms present in the soil is easier. The type of soil is important due to the varying difficulty in cleaning it [122], which may allow for the persistence of transmission forms. As mentioned earlier, this circumstance should be especially considered in sleeping quarters or in isolated themed environments, where microclimates that serve as foci for parasitic infection can develop (of particular relevance in direct life cycle parasites). Among carnivores, the giant anteater is a special case; being in an outdoor installation with natural soil, it can also easily become infected by arthropods or annelids that it preys upon in the enclosure.

### 4.3. Transmission Risks between Animals and Humans

Almost all parasite species identified in this study followed a direct life cycle. The trematode eggs discovered in two samples from bears at ZooAquarium were not identified, thus hindering the evaluation of transmission risk to other animals. The bears were housed in enclosures with minimal vegetation, limiting the presence of snails that could serve as intermediate hosts; however, their enclosure features a safety moat where plants grow, and snails might live there and could potentially continue the parasite cycle. The fact that the eggs were detected only once suggests that the infection was likely due to metacercariae in the supplied food rather than an active ongoing cycle, but a natural infection cannot be ruled out.

The adult stage of cestodes exhibits some host specificity, while the larval stages could frequently affect a wide range of intermediate hosts. The guineafowl releasing *Raillietina*-like eggs would probably become infected after ingesting parasitised insects that freely accessed the bird facilities. This was an exceptional situation, as the finding occurred only once in the last 10 years. In mammals, the cestode eggs found were also detected only once in each positive host species; the fact that the morphology of the eggs does not correspond to any species previously described in the hosts suggests that some or all of them may be spurious parasitoses. Despite the greater abundance of wildlife around ZooAquarium, and until it can be confirmed that they are real parasitoses, there is no evidence for a higher rate of transmission of indirect life cycle parasites in one or the other zoo; however, the possibility of their occurrence exists. In none of the positive cases, the eggs found corresponded to taeniids, which could be the most dangerous cestodes for humans and other mammals as cysticerci could develop in their internal organs and may cause death. Cysticerci were recovered three times in the surgeries or necropsies of some animals at the ZooAquarium in the last 20 years (*T. crassiceps* cysticerci found in 2007 in a ring-tailed lemur [123]; unidentified cysticerci found in 2009 in a dorcas gazelle, and *T. crassiceps* cysticerci found in 2017 in a ring-tailed lemur [124]). The origin of these infections was not established, but at least for the 2007 lemur cysticercosis, it was suspected to have occurred before the animal arrived at the ZooAquarium [123]. However, in the park of origin, the infection would have occurred through any of the routes already mentioned in Section 4.2.

The nematodes found in birds are not infective species for mammals, so they do not pose a risk to zoo staff or visitors, although they can be transmitted to wild birds that enter the facilities seeking food. In the zoos considered in this study, this transmission seems unlikely, as most of the nematodes were detected in birds of prey, and wild fauna would avoid entering the areas within their range. At Faunia, findings were occasional in Galliformes and Gruiformes; the exception being toucans, where *Capillaria* infection recurs over time; however, in this case, the birds are in a closed installation inaccessible to wild avifauna from the area.

Transmission of parasites between zoos is possible due to the exchange of animals that may be parasitised [44]. Pre-transportation analyses of animals or quarantine periods at the receiving zoo are essential to prevent parasite dissemination. Likewise, there is a risk of parasite transmission from zoo animals to wildlife when zoos participate in breeding and species repopulation/reintroduction programs in their original habitats. It has been proposed that pre-exposure to some pathogens (i.e., parasites) can increase host survival rates once released into the wild [24,125,126]. However, zoos can serve as hotspots for gastrointestinal parasites [115,127], and hidden host–parasite co-reintroductions could occur [128]. This can affect both the reintroduced animals and/or the target population [127], leading to difficulties or failure in some reintroduction programs [24]. Husbandry practices are of special relevance to avoid reintroduction of apparently healthy but parasitised animals into wild populations [24,115]. The gastrointestinal parasites that can be involved in the success or failure of reintroduction programs should be considered on a case-by-case basis; in general, coccidia and nematodes would be the most important ones [115,129,130].

In our study, we identified certain parasites that are potentially transmissible between animals and humans (*Entamoeba* spp., *Giardia* spp., *B. coli*, *Trichuris* spp.). In a previous molecular-based investigation [15], there was no evidence of transmission between these parasites and the personnel at ZooAquarium and Faunia; however, zoonotic transmission was detected for *Cryptosporidium hominis* Morgan-Ryan et al. 2002 and *Blastocystis* (Alexeieff 1911) spp. In some studies conducted in other zoos, potential transmission of parasites to zoo personnel was also suggested [11,12,13,14,131,132]. The risk of transmission to visitors is low, as contact with animals is generally limited or nonexistent; however, zookeepers and veterinarians are exposed during handling and cleaning operations. Regular analyses of the animals and a personnel health program incorporating proper training, periodic testing, and health monitoring would minimise transmission risks between animals and caretakers [122].

## 5. Conclusions

The findings of this study largely align with those reported by other authors, indicating that parasites with direct life cycle, including protists and helminths, are predominant in captive animals. Most of the parasite species identified exhibit low or no pathogenicity; the predominance of this type of parasite could be attributed to a combination of factors: (1) Non-pathogen species are typically not investigated or treated in animals, thus facilitating their spread. Conversely, potentially pathogenic species are detected and treated in animals. (2) Animals are fed a controlled diet, which helps prevent or at least limit infections.

Cleaning and disinfecting soil is relatively easy to achieve in artificial (usually concrete) substrates. However, in natural soils, this is often not feasible, and parasites with direct life cycles are difficult to eliminate from the facilities, leading to periodic reinfections. In these circumstances, animals that feed on the ground (e.g., herbivores) have an increased likelihood of becoming infected. This also applies to non-human primates, as they commonly use their hands for locomotion and food manipulation. Regular analyses and preventive/therapeutic antiparasitic treatments would be the optimal approach to maintaining a low intensity of parasite infections and to reduce the risk of zoonotic transmission.

## Figures and Tables

**Table 1:** Total number of mammalian and avian species analysed at ZooAquarium and Faunia zoological parks, Madrid, Spain.

Zoo	Hosts	Diet Type	Animal Species Studied	Hosts Infected	Samples Analysed	Positive Samples
ZooAquarium	Mammals	Herbivores	55	49 (89.1%)	1891	956 (50.6%)
		Omnivores	17	9 (53.0%)	455	164 (36.0%)
		Carnivores	11	2 (18.2%)	254	27 (10.6%)
		Total	83	60 (72.3%)	2600	1147 (44.1%)
	Birds	Herbivores	17	0 (0.0%)	127	0 (0.0%)
		Omnivores	15	3 (20.0%)	127	8 (6.3%)
		Carnivores	32	4 (12.5%)	285	26 (9.1%)
		Total	64	7 (11.0%)	539	34 (6.3%)
Faunia	Mammals	Herbivores	29	17 (58.6%)	783	136 (17.4%)
		Omnivores	23	7 (30.4%)	671	46 (6.9%)
		Carnivores	16	1 (6.3%)	422	2 (0.5%)
		Total	68	25 (36.8%)	1876	184 (9.8%)
	Birds	Herbivores	14	0 (0.0%)	130	0 (0.0%)
		Omnivores	12	5 (41.7%)	188	29 (15.4%)
		Carnivores	14	0 (0.0%)	94	0 (0.0%)
		Total	40	5 (12.5%)	412	29 (7.0%)
Total results *	Mammals	Herbivores	73	62 (84.9%)	2674	1092 (40.8%)
		Omnivores	34	16 (47.1%)	1126	211 (18.7%)
		Carnivores	25	4 (16.0%)	676	30 (4.4%)
		Total	132	82 (62.1%)	4476	1333 (29.8%)
	Birds	Herbivores	24	0 (0.0%)	257	0 (0.0%)
		Omnivores	22	7 (31.8%)	315	37 (11.8%)
		Carnivores	40	4 (10.0%)	379	26 (6.9%)
		Total	86	11 (12.8%)	951	63 (6.6%)

* The number of host species does not correspond to the direct sum of species from both zoos, as there are 19 species of mammals and 18 of birds housed in both centres.

**Table 2:** Number of mammal and avian species found infected by different parasites in each zoological centre. Codes: C—carnivorous, O—omnivorous, H—herbivorous. The number in the parenthesis under the code indicates the number of host species for each classification.

		ZooAquarium							Faunia					
		Infected Host Species							Infected Host Species					
		Mammals			Birds				Mammals			Birds		
		C (2)	O (9)	H (49)	C (4)	O (3)	H (0)		C (1)	O (7)	H (17)	C (0)	O (5)	H (0)
*Amoebae*														
	*Entamoeba* Casagrandi and Barbagallo 1897	1	6	34		3				3	9		1	
	*Endolimax* Kuenen and Swellengrebel 1913		2	6							2			
*Flagellates*														
	*Giardia* Künstler 1882		1	4						1	3			
	*Chilomastix* Aléxéieff 1910		3	8						2	2			
	Trichomonads	1		1										
*Coccidia*														
	*Eimeria* Schneider 1875			5							2			
	*Toxoplasma* Nicolle and Manceaux 1909/*Neospora* Dubey et al. 1988	1												
*Ciliates*														
	*Balantioides* Alexeieff 1931		5	8		1				2	1			
	*Buxtonella* Jameson 1926			5										
	*Troglodytella* (Brumpt and Joyeux 1912)			1										
	Endosymbiotic ciliates			6							2			
*Trematodes*														
	Unidentified eggs		1											
*Cestodes*														
	Unidentified eggs		1	5										1
*Nematodes*														
	*Trichuris* Roederer 1761		3	10	1				1	1	6			
	*Capillaria* Zeder 1800/capillariids	1	1	2	3						1		3	
	*Nematodirus* Ransom 1907			2										
	*Trichostrongylids*			1										
	*Baylisascaris* Sprent 1968		1							1				
	*Parascaris* Yorke and Maplestone 1926			2										
	*Porrocaecum* Railliet and Henry 1912				1									
	*Ascaridia* Dujardin 1845/*Heterakis* Schrank 1790												2	
	Ascarid (unidentified)								1					

**Table 3:** List of parasites found in the mammal hosts at ZooAquarium. Species in bold are also housed at Faunia Park.

			Samples	
Order	Family	Species	(Total/Positives)	Parasites Found (% of Total Samples)
Artiodactyla	Bovidae	*Ammotragus lervia* (Pallas 1777)	21/14	*Entamoeba bovis*-like (66.7%)
		*Antilope cervicapra* (Linnaeus 1758)	18/9	*Entamoeba bovis*-like (50.0%)
		*Bison bison* (Linnaeus 1758)	37/15	*Entamoeba bovis*-like (40.5%)
		*Bison bonasus* (Linnaeus 1758)	27/19	*Entamoeba bovis*-like (70.4%), *Buxtonella sulcata* Jameson 1926 (3.7%)
		*Bos grunniens* (Linnaeus 1766)	17/12	*Entamoeba bovis*-like (52.9%), *Buxtonella sulcata* (41.2%), unidentified cestode eggs (5.9%)
		*Bos taurus* (Linnaeus 1758)	4/1	*Entamoeba bovis*-like (25.0%)
		*Boselaphus tragocamelus* (Pallas 1766)	13/12	*Entamoeba bovis*-like (92.3%)
		*Budorcas taxicolor* Hodgson 1850	39/26	*Entamoeba bovis*-like (66.7%)
		***Capra hircus*** **Linnaeus 1758**	37/31	*Entamoeba bovis*-like (81.1%), *Eimeria* spp. (5.4%), *Trichuris* spp. (5.4%)
		*Capra pyrenaica* Schinz 1838	30/20	*Entamoeba bovis*-like (66.7%)
		*Connochaetes gnou* (Zimmermann 1780)	23/14	*Entamoeba bovis*-like (60.9%)
		*Gazella dorcas osiris* Blaine 1913	127/106	*Entamoeba bovis*-like (70.1%), *Eimeria* spp. (1.6%), *Trichuris* spp. (16.5%), *Nematodirus* spp. (21.3%)
		*Nanger dama mhorr* (Bennett 1833)	99/85	*Entamoeba bovis*-like (74.7%), *Entamoeba* spp. (8-nucleated) (2.0%), *Giardia* spp. (2.0%), *Chilomastix* spp. (1.0%), *Eimeria* spp. (2.0%), *Trichuris* spp. (15.2%), *Nematodirus* spp. (8.1%), Trichostrongylids (10.1%)
		***Ovis aries*** **Linnaeus 1758**	39/13	*Entamoeba bovis*-like (25.6%), *Chilomastix* spp. (2.6%), *Eimeria* spp. (5.1%)
		*Ovis gmelinii* Blyth 1841	11/6	*Entamoeba bovis*-like (54.6%)
		*Syncerus caffer nanus* Boddaert 1785	59/54	*Entamoeba bovis*-like (89.8%), trichomonads (3.4%), *Buxtonella sulcata* (44.1%)
		*Tragelaphus eurycerus* (Ogilby 1837)	17/15	*Entamoeba bovis*-like (88.2%)
		*Tragelaphus spekii gratus* Sclater 1880	40/26	*Entamoeba bovis*-like (60.0%), *Chilomastix* spp. (2.5%), *Balantioides coli*-like (15.0%)
	Camelidae	*Camelus bactrianus* Linnaeus 1758	40/19	*Entamoeba bovis*-like (20.0%), *Buxtonella cameli* (Boschenko 1925) (25.0%), *Trichuris* spp. (5.0%)
		***Camelus dromedarius*** **Linnaeus 1758**	34/7	*Entamoeba bovis*-like (5.9%), *Buxtonella cameli* (20.6%)
		***Lama guanicoe*** **(Müller 1776)**	19/8	*Entamoeba bovis*-like (42.1%)
	Cervidae	*Alces alces* (Linnaeus 1758)	16/11	*Balantioides coli*-like (43.8%), *Trichuris* spp. (56.3%)
		*Capreolus capreolus* (Linnaeus 1758)	6/5	*Entamoeba bovis*-like (83.3%)
		***Cervus elaphus*** **Linnaeus 1758**	34/30	*Entamoeba bovis*-like (85.3%), unidentified cestode eggs (2.9%)
		*Dama dama* (Linnaeus 1758)	40/35	*Entamoeba bovis*-like (87.5%), *Capillaria* spp. (5.0%)
		*Elaphurus davidianus* Milne-Edwards 1866	29/20	*Entamoeba bovis*-like (69.0%)
		*Muntiacus reevesi* (Ogilby 1839)	21/18	*Entamoeba bovis*-like (85.7%), *Chilomastix* spp. (4.8%)
		*Rangifer tarandus* (Linnaeus 1758)	23/4	*Entamoeba bovis*-like (4.4%), *Trichuris* spp. (13.0%)
	Giraffidae	*Giraffa camelopardalis* (Linnaeus 1758)	40/14	*Entamoeba bovis*-like (35.0%), *Trichuris* spp. (2.5%)
	Hippopotamidae	*Hippopotamus amphibius* Linnaeus 1758	11/1	Unidentified cestode eggs (9.1%)
	Suidae	*Potamochoerus porcus* (Linnaeus 1758)	37/14	*Entamoeba polecki*-like (29.7%), *Giardia* spp. (2.7%), *Chilomastix* spp. (8.1%), *Balantioides coli* (24.3%)
		***Sus scrofa*** **Linnaeus 1758**	59/43	*Entamoeba polecki*-like (52.5%), *Chilomastix* spp. (8.5%), *Balantioides coli* (42.4%)
	Tayassuidae	***Dicotyles tajacu*** **(Linnaeus 1758)**	1/0	
Carnivora	Ailuridae	***Ailurus fulgens*** **Cuvier 1825**	33/2	*Capillaria* spp. (6.1%)
	Canidae	*Canis lupus occidentalis* Linnaeus 1758	15/0	
		*Speothos venaticus* (Lund 1842)	45/1	*Toxoplasma/Neospora* (2.2%)
	Felidae	*Lynx lynx* (Linnaeus 1758)	13/0	
		*Lynx pardinus* (Temminck 1827)	20/0	
		*Panthera leo* (Linnaeus 1758)	16/0	
		*Panthera pardus saxicolor* (Linnaeus 1758)	22/0	
		*Panthera tigris* (Linnaeus 1758)	16/0	
	Herpestidae	***Suricata suricatta*** **(Schreber 1776)**	10/0	
	Mustelidae	***Mustela lutreola*** **(Linnaeus 1761)**	30/0	
		*Pteronura brasiliensis* (Zimmermann 1780)	20/0	
	Procyonidae	***Nasua nasua*** **(Linnaeus 1766)**	9/0	
		***Procyon lotor*** **(Linnaeus 1758)**	18/2	*Capillaria* spp. (11.1%)
	Ursidae	*Ailuropoda melanoleuca* (David 1869)	36/0	
		*Helarctos malayanus* (Raffles 1822)	35/1	Trematoda (2.9%)
		*Tremarctos ornatus* (Cuvier 1825)	1/0	
		*Ursus americanus* Pallas 1780	16/0	
		*Ursus arctos* Linnaeus 1758	63/7	*Baylisascaris* spp. (11.1%)
		*Ursus thibetanus* Cuvier 1823	37/0	
	Viverridae	***Arctictis binturong*** **(Raffles 1822)**	32/0	
Diprotodontia	Macropodidae	***Notamacropus rufogriseus*** **(Desmarest 1817)**	6/0	
		*Petrogale xanthopus* Gray 1855	20/5	*Entamoeba bovis*-like (25.0%)
	Phascolarctidae	*Phascolarctos cinereus* (Goldfuss 1817)	35/0	
Lagomorpha	Leporidae	***Oryctolagus cuniculus*** **(Linnaeus 1758)**	65/4	*Eimeria* spp. (6.2%)
Perissodactyla	Equidae	*Equus quaga* Boddaert 1785	159/9	Endosymbiotic ciliates (4.4%), *Parascaris equorum* (Goeze 1782) (1.3%)
		*Equus asinus* Linnaeus 1758	25/21	Endosymbiotic ciliates (84.0%), *Parascaris equorum* (8.0%)
		***Equus caballus*** **Linnaeus 1758**	44/28	Endosymbiotic ciliates (63.6%)
	Rhinocerotidae	*Ceratotherium simum* (Burchell 1817)	35/18	Endosymbiotic ciliates (51.4%)
		*Rhinoceros unicornis* Linnaeus 1758	32/27	Endosymbiotic ciliates (84.4%)
	Tapiridae	*Tapirus indicus* (Desmarest 1819)	31/1	*Chilomastix* spp. (3.2%)
		*Tapirus terrestris* (Linnaeus 1758)	12/2	*Balantioides coli* (8.3%), unidentified cestode eggs (8.3%)
Pilosa	Myrmecophagidae	*Myrmecophaga tridactyla* Linnaeus 1758	47/26	*Entamoeba* spp. (4-nucleated) (2.1%), *Tetratrichomonas* spp. Parisi 1910 (25.5%), *Capillaria*-like eggs (36.2%)
Primates	Cebidae	***Sapajus apella*** **(Linnaeus 1758)**	26/0	
	Cercopithecidae	*Colobus guereza* Rüppell 1835	45/40	*Entamoeba coli*-like (24.4%), *Entamoeba polecki*-like (22.2%), *Balantioides coli*-like (2.2%), *Trichuris* spp. (84.4%)
		*Macaca* spp. Lacepede 1799	1/0	
		*Mandrillus sphinx* (Linnaeus 1758)	47/47	*Entamoeba polecki*-like (76.6%), *Entamoeba coli*-like (10.6%), *Chilomastix* spp. (10.6%), *Balantioides coli*-like (66.0%), *Trichuris* spp. (2.1%), unidentified cestode eggs (2.1%)
		*Papio* spp. Erxleben 1777	17/17	*Entamoeba coli*-like (94.1%), *Endolimax* spp. (5.9%), *Trichuris* spp. (82.4%)
	Hominidae	*Pongo pygmaeus* (Linnaeus 1760)	103/79	*Balantioides coli*-like (76.7%)
		*Gorilla gorilla* (Savage 1847)	29/17	*Entamoeba coli*-like (3.5%), *Balantioides coli*-like (51.7%), *Troglodytella abrassarti* (Brumpt and Joyeux 1912) (10.3%)
		*Pan troglodytes* (Blumenbach 1775)	28/21	*Entamoeba coli*-like (39.3%), *Entamoeba polecki*-like (14.3%), *Endolimax* spp. (3.6%), *Balantioides coli*-like (39.3%)
	Hylobatidae	*Hylobates lar* (Linnaeus 1771)	25/9	*Entamoeba coli*-like (4.0%), *Entamoeba polecki*-like (4.0%), *Balantioides coli*-like (28.0%)
		*Hylobates muelleri* (Martin 1841)	28/12	*Entamoeba coli*-like (28.6%), *Balantioides coli*-like (14.3%), *Trichuris* spp. (3.6%)
	Lemuridae	*Eulemur fulvus* (Geoffroy 1796)	14/2	*Giardia* spp. (7.1%), unidentified cestode eggs (7.1%)
		***Lemur catta*** Linnaeus 1758	16/2	*Entamoeba polecki*-like (6.3%), *Giardia* spp. (6.3%), *Trichuris* spp. (6.3%)
		***Varecia variegata*** **(Kerr 1792)**	40/0	
Proboscidea	Elephantidae	*Elephas maximus* Linnaeus 1758	55/30	*Chilomastix* spp. (1.8%), endosymbiotic ciliates (54.5%)
Rodentia	Caviidae	***Cavia porcellus*** (Linnaeus 1758)	17/0	
		***Dolichotis patagonum*** **(Zimmermann 1780)**	27/8	*Entamoeba muris*-like (3.7%), *Giardia* spp. (14.8%), *Chilomastix* spp. (7.4%), *Trichuris* spp. (3.7%)
		***Hydrochoerus hydrochaeris*** **(Linnaeus 1766)**	15/2	*Chilomastix* spp. (6.7%), *Balantioides coli*-like (6.7%)
	Chinchillidae	*Chinchilla* spp. Bennett 1829	1/0	

**Table 4:** List of parasites found in the avian hosts at ZooAquarium. Species in bold are also housed at Faunia Park.

			Samples	
Order	Family	Species	(Total/Positives)	Parasites Found (% of Total Samples)
Accipitriformes	Accipitridae	***Aegypius monachus*** **(Linnaeus 1766)**	7/0	
		*Aquila adalberti* Brehm 1861	13/0	
		*Aquila verreauxii* Lesson 1831	3/0	
		*Buteo buteo* (Linnaeus 1758)	13/0	
		***Geranoaetus melanoleucus*** **(Vieillot 1819)**	3/0	
		*Gypohierax angolensis* (Gmelin 1788)	2/0	
		***Gyps fulvus*** **(Hablizl 1783)**	23/0	
		*Haliaeetus albicilla* (Linnaeus 1758)	3/0	
		***Haliaeetus leucocephalus*** **(Linnaeus 1766)**	3/0	
		***Haliaeetus pelagicus*** **(Pallas 1811)**	10/3	Capillariids (34.3%)
		*Ichtyophaga vocifer* (Daudin 1800)	3/0	
		*Milvus migrans* (Boddaert 1783)	36/21	Capillariids (38.9%), *Porrocaecum* spp. (33.3%)
		*Neophron percnopterus* (Linneaus 1758)	3/0	
		***Parabuteo unicinctus*** **(Temminck 1824)**	28/1	*Trichuris* spp. (3.6%)
Anseriformes	Anatidae	*Alopochen aegyptiaca* (Linnaeus 1766)	5/0	
		*Aythya nyroca* (Güldenstädt 1770)	1/0	
		*Cairina moschata* (Linnaeus 1758)	8/0	
		***Cygnus atratus*** **(Latham 1790)**	1/0	
		*Tadorna ferruginea* (Pallas 1764)	4/0	
		*Tadorna tadorna* (Linnaeus 1758)	3/0	
Bucerotiformes	Bucerotidae	*Bycanistes brevis* Friedmann 1929	5/0	
		*Bycanistes bucinator* (Temminck 1824)	8/0	
	Bucorvidae	*Bucorvus leadbeateri* (Vigors 1825)	5/0	
Cathartiformes	Cathartidae	*Sarcoramphus papa* (Linnaeus 1758)	10/0	
		*Vultur gryphus* Linnaeus 1758	5/0	
Ciconiiformes	Ciconiidae	*Ciconia ciconia* (Linnaeus 1758)	10/0	
		*Leptoptilos crumenifer* (Lesson 1831)	3/0	
Columbiformes	Columbidae	*Columba livia* Gmelin 1789	11/0	
Coraciiformes	Alcedinidae	*Dacelo novaeguineae* Hermann 1783	2/0	
Falconiformes	Falconidae	*Falco naumanni* Fleischer 1818	13/0	
		*Caracara plancus* (Miller 1777)	1/0	
Galliformes	Numididae	***Numida meleagris*** **(Linnaeus 1758)**	4/0	
	Phasianidae	*Gallus gallus* (Linnaeus 1758)	39/1	*Entamoeba gallinarum* Tyzzer 1920 (2.6%)
Gruiformes	Gruidae	*Balearica regulorum* (Bennett 1834)	4/0	
Musophagiformes	Musophagidae	*Tauraco erythrolophus* (Vieillot 1819)	8/0	
		***Menelikornis leucotis*** **(Rüppell 1835)**	5/0	
Passeriformes	Corvidae	*Corvus corax* Linnaeus 1758	3/0	
Pelecaniformes	Pelecanidae	***Pelecanus rufescens*** Gmelin 1789	8/0	
	Threskiornithidae	*Eudocimus ruber* (Linnaeus 1758)	11/0	
		*Threskiornis aethiopicus* (Latham 1790)	23/0	
Phoenicopteriformes	Phoenicopteridae	***Phoenicopterus ruber*** **Linnaeus 1758**	7/0	
Piciformes	Ramphastidae	***Ramphastos toco*** **Müller 1776**	5/0	
Psittaciformes	Cacatuidae	*Cacatua alba* (Müller 1776)	6/0	
		*Cacatua galerita* (Latham 1790)	11/0	
		*Cacatua goffiniana* Roselaar and Michels 2004	5/0	
		*Cacatua pastinator* (Gould 1841)	8/0	
		*Cacatua sulphurea* (Gmelin 1788)	5/0	
	Psittacidae	***Amazona aestiva*** **(Linnaeus 1758)**	8/0	
		***Ara ararauna*** **(Linnaeus 1758)**	22/0	
		***Ara chloropterus*** **Gray 1859**	8/0	
		***Ara rubrogenys*** **Lafresnaye 1847**	1/0	
		***Aratinga solstitialis*** **(Linnaeus 1758)**	15/0	
		*Myiopsitta monachus* Boddaert 1783	1/0	
		*Psittacus erithacus* Linnaeus 1758	7/0	
		*Trichoglossus haematodus* (Linnaeus 1771)	4/0	
	Psittaculidae	***Eclectus roratus*** **(Müller 1776)**	2/0	
Strigiformes	Strigidae	***Bubo bubo hispanus*** **Rothschild and Hartert 1910**	15/1	Capillariids (6.7%)
		***Bubo bubo sibiricus*** **Gloger 1833**	5/0	
		*Bubo scandiacus* (Linnaeus 1758)	2/0	
		*Strix nebulosa* Forster 1772	1/0	
Casuariformes	Casuariidae	*Casuarius casuarius* (Linnaeus 1758)	12/0	
Struthioniformes	Dromaiidae	***Dromaius novaehollandiae*** **(Latham 1790)**	20/0	
Rheiformes	Rheidae	***Rhea americana*** **(Linnaeus 1758)**	7/2	*Entamoeba* spp. (4-nucleated) (28.6%)
Struthioniformes	Struthionidae	*Struthio camelus* Linnaeus 1758	12/5	*Entamoeba polecki*-like (33.3%), *Balantioides coli* (8.3%)

**Table 5:** List of parasites found in the mammal hosts at Faunia Park. Species in bold are also housed at ZooAquarium.

			Samples	
Order	Family	Species	(Total/Positives)	Parasites Found (% of Total Samples)
Afrosoricida	Tenrecidae	*Echinops telfairi* Martin 1838	2/0	
Artiodactyla	Bovidae	***Capra hircus***	40/22	*Entamoeba bovis*-like (55.0%), *Eimeria* spp. (2.5%)
		*Madoqua kirkii* (Günther 1880)	43/15	*Entamoeba bovis*-like (11.6%), *Entamoeba* spp. (8-nucleated) (27.9%), *Trichuris* spp. (2.3%)
		*Ovis aries*	26/10	*Entamoeba bovis*-like (34.6%), *Eimeria* spp. (3.9%)
	Cervidae	*Subulo gouazoubira* (Fischer 1814)	12/11	*Entamoeba bovis*-like (91.7%), *Trichuris* spp. (8.3%)
		*Muntiacus muntjack* Zimmermann 1780	47/18	*Entamoeba bovis*-like (36.2%), *Entamoeba* spp. (8-nucleated) (2.1%), *Giardia* spp. (2.1%), *Trichuris* spp. (4.3%)
	Suidae	***Sus scrofa***	39/22	*Entamoeba polecki*-like (46.2%), *Chilomastix* spp. (7.7%), *Balantioides coli* (28.2%)
	Tayassuidae	***Dicotyles tajacu***	16/1	*Balantioides coli* (6.3%)
Carnivora	Ailuridae	***Ailurus fulgens***	36/0	
	Canidae	*Vulpes zerda* (Zimmermann 1780)	52/2	*Trichuris* spp. (1.9%), unidentified ascarid (1.9%)
	Felidae	*Leopardus pardalis* (Linnaeus 1758)	48/0	
	Herpestidae	*Helogale parvula* (Sundevall 1847)	32/0	
		***Suricata suricatta***	15/0	
	Mephitidae	*Mephitis mephitis (*Schreber 1776)	50/3	*Baylisascaris* spp. (6.0%)
	Mustelidae	***Mustela lutreola***	19/0	
		*Mustela putorius furo* Linnaeus 1758	5/0	
	Procyonidae	***Nasua nasua***	45/0	
		*Potos flavus* (Schreber 1774)	49/0	
		***Procyon lotor***	30/0	
	Viverridae	***Arctictis binturong***	25/0	
		*Genetta genetta* (Linnaeus 1758)	45/0	
Chiroptera	Phyllostomidae	*Carollina perspicillata* (Linnaeus 1758)	13/0	
	Pteropodidae	*Rousettus aegyptiacus* (Saint-Hilaire 1810)	26/0	
Cingulata	Chlamyphoridae	*Euphractus sexcinctus* (Linnaeus 1758)	16/0	
		*Chaetophractus villosus* (Desmarest 1804)	16/0	
		*Tolypeutes tricinctus* (Linnaeus 1758)	2/0	
Dasyurimorpha	Dasyuridae	*Dasyurus viverrinus* (Shaw 1800)	24/0	
Diprotodontia	Macropodidae	***Notamacropus rufogriseus***	46/4	*Entamoeba* spp. (one nucleated) (6.5%), *Entamoeba* spp. (8-nucleated) (2.2%)
		*Osphranter rufus* (Desmarest 1822)	42/0	
Eulipotyphla	Erinaceidae	*Atelerix albiventris* (Wagner 1841)	35/0	
Lagomorpha	Leporidae	***Oryctolagus cuniculus***	1/0	
Perissodactyla	Equidae	*Equus africanus* (Heuglin and Fitzinger 1866)	27/9	Endosymbiotic ciliates (33.3%)
		***Equus caballus***	35/8	Endosymbiotic ciliates (22.9%)
Pilosa	Choloepodidae	*Choloepus didactilus* (Linnaeus 1758)	46/13	*Entamoeba* spp. (8-nucleated) (28.3%)
	Myrmecophagidae	*Tamandua tetradactyla* (Linnaeus 1758)	36/0	
Primates	Aotidae	*Aotus nancymaae* Hershkovitz 1983	7/1	*Entamoeba coli*-like (14.3%)
		*Aotus trivirgatus* (Humboldt 1812)	9/0	
	Callitrichidae	*Callimico goeldii* (Thomas 1904)	47/0	
		*Callithrix jacchus* (Linnaeus 1758)	38/0	
		*Cebuella pygmaea* (Spix 1823)	11/0	
		*Leontopithecus rosalia* (Linnaeus 1766)	31/0	
		*Saguinus geoffroyi* (Pucheran 1845)	30/0	
		*Saguinus imperator* (Goeldi 1907)	34/0	
		*Saguinus oedipus* (Linnaeus 1758)	26/0	
	Cebidae	***Sapajus apella***	30/0	
		*Saimiri sciureus* (Linnaeus 1758)	53/0	
	Galagidae	*Galago moholi* Smith 1836	19/0	
	Lemuridae	*Eulemur albifrons* (Geoffroy 1796)	17/0	
		***Lemur catta***	26/0	
		***Varecia variegata***	3/0	
		*Varecia rubra* (Geoffroy 1812)	12/1	*Capillaria* spp. (8.3%)
	Lorisidae	*Xanthonycticebus pygmaeus* (Bonhote 1907)	5/0	
		*Perodicticus potto* (Müller 1766)	26/0	
	Pitheciidae	*Pithecia pithecia* (Linnaeus 1766)	33/2	*Entamoeba coli*-like (6.1%)
Rodentia	Heterocephalidae	*Heterocephalus glaber* Rüppell 1842	29/0	
	Caviidae	***Cavia porcellus***	54/2	*Balantioides coli* (3.7%)
		***Dolichotis patagonum***	24/7	*Giardia* spp. (8.3%), *Trichuris* spp. (20.8%)
		***Hydrochoerus hydrochaeris***	1/0	
	Dasyproctidae	*Dasyprocta azarae* Lichtenstein 1823	1/0	
		*Dasyprocta fuliginosa* Wagler 1832	23/3	*Trichuris* spp. (13.0%)
		*Dasyprocta punctata* Gray 1842	8/0	
	Dipodidae	*Jaculus orientalis* Erxleben 1777	12/3	*Entamoeba muris* (25.0%), *Chilomastix* spp. (16.7%)
	Echimyidae	*Capromys pilorides* (Say 1822)	94/15	*Trichuris* spp. (16.0%)
	Erethizontidae	*Coendou prehensilis* (Linnaeus 1758)	43/5	*Chilomastix* spp. (9.3%), *Trichuris* spp. (2.3%)
	Hystricidae	*Hystrix cristata* Linnaeus 1758	47/5	*Entamoeba* spp. (8-nucleated) (2.1%), *Giardia* spp. (8.5%)
	Pedetidae	*Pedetes capensis* (Forster 1778)	31/0	
	Sciuridae	*Cynomys ludovicianus* (Ord 1815)	4/1	*Chilomastix* spp. (25.0%)
Tubulidentata	Orycteropodidae	*Orycteropus afer* (Pallas 1766)	7/1	*Giardia* spp. (14.3%)

**Table 6:** List of parasites found in the avian hosts at Faunia Park. Species in bold are also housed at ZooAquarium.

			Samples	
Order	Family	Species	(Total/Positives)	Parasites Found (% of Total Samples)
Accipitriformes	Accipitridae	***Necrosyrtes monachus***	8/0	
		*Aquila nipalensis* Hodgson 1833	5/0	
		*Buteo jamaicensis* (Gmelin 1788)	6/0	
		*Buteo regalis* (Gray 1844)	5/0	
		***Geranoaetus melanoleucus***	13/0	
		***Gyps fulvus***	7/0	
		***Parabuteo unicinctus***	14/0	
Anseriformes	Anatidae	***Cygnus atratus***	5/0	
	Anhimidae	*Chauna torquata* (Oken 1816)	11/0	
Charadriiformes	Recurvirostridae	*Recurvirostra avosetta* Linnaeus 1758	1/0	
Falconiformes	Falconidae	*Phalcoboenus australis* (Gmelin 1788)	2/0	
Galliformes	Numididae	***Numida meleagris***	7/3	*Entamoeba gallinarum* (14.3%), capillariids (14.3%)*, Ascaridia* spp.*/Heterakis* spp. (28.6%), *Raillietina*-like eggs (14.3%)
	Phasianidae	***Gallus gallus***	14/1	*Ascaridia* spp./*Heterakis* spp. (7.1%)
		*Meleagris gallopavo* Linnaeus 1758	39/0	
Gruiformes	Gruidae	*Grus grus* (Linnaeus 1758)	6/1	Capillariids (16.7%)
		*Grus virgo* (Linnaeus 1758)	8/0	
Musophagiformes	Musophagidae	***Menelikornis leucotis***	11/0	
Passeriformes	Corvidae	*Calocitta formosa* (Swainson 1827)	3/0	
	Cotingidae	*Rupicola peruvianus* (Latham 1790)	22/0	
	Sturnidae	*Lamprotornis purpureus* (Müller 1776)	2/0	
Pelecaniformes	Ardeidae	*Bubulcus ibis* (Linnaeus 1758)	1/0	
	Pelecanidae	*Pelecanus onocrotalus* Linnaeus 1758	2/0	
Phoenicopteriformes	Phoenicopteridae	***Phoenicopterus ruber***	5/0	
Piciformes	Ramphastidae	*Ramphastos swainsonii* Gould, 1833	21/17	*Capillaria* spp. (81.0%)
		***Ramphastos toco***	16/7	*Capillaria* spp. (43.8%)
Psittaciformes	Cacatuidae	*Eolophus roseicapilla* (Vieillot 1817)	5/0	
	Psittacidae	***Amazona aestiva***	27/0	
		***Ara ararauna***	17/0	
		***Ara chloropterus***	6/0	
		*Ara macao* (Linnaeus 1758)	3/0	
		*Ara militaris* (Linnaeus 1766)	1/0	
		***Ara rubrogenys***	2/0	
		***Aratinga solstitialis***	9/0	
	Psittaculidae	***Eclectus rotarus***	8/0	
		*Trichoglossus haematodus* (Linnaeus 1771)	3/0	
Strigiformes	Strigidae	***Bubo bubo hispanus***	9/0	
		***Bubo bubo sibiricus***	6/0	
	Tytonidae	*Tyto alba* Scopoli 1769	11/0	
Casuariiformes	Casuariidae	***Dromaius novaehollandiae***	48/0	
Rheiformes	Rheidae	***Rhea americana***	23/0	

**Table 7:** Number of positive and total (in parenthesis) mammalian and avian species analysed according to their housing conditions and feeding habits.

			Zoological Park												
			ZooAquarium							Faunia					
			Feeding Habits							Feeding Habits					
Host Class	Isolation	Soil	Carnivorous		Omnivorous		Herbivorous			Carnivorous		Omnivorous		Herbivorous	
Mammal	accessible	Natural	2	(10)	8	(13)	48	(53)		0	(1)	2	(3)	12	(19)
		Artificial	0	(0)	0	(0)	1	(1)		0	(0)	0	(0)	0	(0)
		Mixed	1	(1)	1	(2)	0	(1)		0	(0)	0	(0)	0	(0)
	isolated	Natural	0	(0)	0	(0)	0	(0)		0	(0)	0	(0)	0	(0)
		Artificial	0	(0)	1	(2)	0	(0)		1	(14)	5	(20)	5	(10)
		Mixed	0	(0)	0	(0)	0	(0)		0	(1)	0	(0)	0	(0)
Bird	accesible	Natural	0	(8)	3	(12)	0	(2)		0	(3)	3	(10)	0	(3)
		Artificial	0	(0)	0	(0)	0	(0)		0	(0)	0	(0)	0	(1)
		Mixed	4	(24)	0	(3)	0	(15)		0	(11)	2	(2)	0	(10)
	isolated	Natural	0	(0)	0	(0)	0	(0)		0	(0)	0	(0)	0	(0)
		Artificial	0	(0)	0	(0)	0	(0)		0	(0)	0	(0)	0	(0)
		Mixed	0	(0)	0	(0)	0	(0)		0	(0)	0	(0)	0	(0)

**Table 8:** Values and statistical significance of the regression coefficients obtained after including 5 independent variables in the binary logistic regression conducted with the results of the parasitological survey of the mammals and birds at two zoological institutions (ZooAquarium and Faunia) in Madrid, Spain. The dependent variable is “at least once infected”/“never infected”.

		Function Coefficients			Wald’s X^2^ Test		
			Standard Error			Degrees of Freedom	
Parameter		B			Score		Significance
Feeding type					14.733	2	<0.001
	Omnivorous vs. carnivorous	1.581	0.532		8.824	1	0.003
	Herbivorous vs. carnivorous	1.911	0.501		14.555	1	<0.001
Soil type					2.401	2	0.301
	artificial vs. natural	0.063	1.710		0.001	1	0.971
	mixed vs. natural	−0.878	0.572		2.352	1	0.125
Host Class (bird vs. mammal)		−2.103	0.480		19.150	1	<0.001
Zoological institution (Faunia vs. ZooAquarium)		−0.649	0.405		2.567	1	0.109
Isolation type (isolated vs. accessible)		−1.326	1.777		0.577	1	0.456
Constant		−1.639	0.389		17.703	1	<0.001

**Table 9:** Values and statistical significance of the regression coefficients obtained after including 4 independent variables in the binary logistic regression conducted with the results of the parasitological survey in mammals at two zoological institutions (ZooAquarium and Faunia) in Madrid, Spain. The dependent variable is “at least once infected”/“never infected”.

		Function Coefficients			Wald’s X^2^ Test		
Parameter		B	Standard Error		Score	Degrees of Freedom	Significance
Feeding type					21.061	2	<0.001
	Omnivorous vs. carnivorous	2.057	0.780		7.571	1	0.006
	Herbivorous vs. carnivorous	3.187	0.716		19.799	1	<0.001
Soil type					2.453	2	0.293
	artificial vs. natural	18.336	25,170.708		0.000	1	0.999
	mixed vs. natural	−2.016	1.287		2.453	1	0.117
Zoological institution (Faunia vs. ZooAquarium)		−1.245	0.531		5.496	1	0.019
Isolation type (isolated vs. accessible)		−18.995	25,170.708		0.000	1	0.999
Constant		−4.146	4195.118		0.000	1	0.999

**Table 10:** Values and statistical significance of the regression coefficients obtained after including 3 independent variables in the binary logistic regression conducted with the results of the parasitological survey of birds at two zoological institutions (ZooAquarium and Faunia) in Madrid, Spain. The dependent variable is “at least once infected”/“never infected”.

		Function Coefficients			Wald’s X^2^ Test		
Parameter		B	Standard Error		Score	Degrees of Freedom	Significance
Feeding type					5.613	2	0.060
	Omnivorous vs. carnivorous	2.107	0.889		5.613	1	0.018
	Herbivorous vs. carnivorous	−18.913	7283.654		0.000	1	0.998
Soil type					1.403	2	0.496
	artificial vs. natural	0.833	40,847.685		0.000	1	1.000
	mixed vs. natural	1.030	0.870		1.403	1	0.236
Zoological institution (Faunia vs. ZooAquarium)		0.121	0.684		0.031	1	0.860
Constant		−8.164	13,397.685		0.000	1	1.000

## Data Availability

The original contributions presented in the study are included in the article; further inquiries can be directed to the corresponding author.
